# Auranofin exerts antibacterial activity against *Neisseria gonorrhoeae* in a female mouse model of genital tract infection

**DOI:** 10.1371/journal.pone.0266764

**Published:** 2022-04-21

**Authors:** Ahmed E. M. Elhassanny, Nader S. Abutaleb, Mohamed N. Seleem

**Affiliations:** 1 Department of Biomedical Sciences and Pathobiology, Virginia-Maryland College of Veterinary Medicine, Virginia Polytechnic Institute and State University, Blacksburg, Virginia, United States of America; 2 Center for Emerging, Zoonotic and Arthropod-borne Pathogens, Virginia Polytechnic Institute and State University, Blacksburg, Virginia, United States of America; South Dakota State University, UNITED STATES

## Abstract

*Neisseria gonorrhoeae* has been classified by the U.S. Centers for Disease Control and Prevention as an urgent threat due to the rapid development of antibiotic resistance to currently available antibiotics. Therefore, there is an urgent need to find new antibiotics to treat gonococcal infections. In our previous study, the gold-containing drug auranofin demonstrated potent *in vitro* activity against clinical isolates of *N*. *gonorrhoeae*, including multidrug-resistant strains. Therefore, the aim of this study was to investigate the *in vivo* activity of auranofin against *N*. *gonorrhoeae* using a murine model of vaginal infection. A significant reduction in *N*. *gonorrhoeae* recovered from the vagina was observed for infected mice treated with auranofin compared to the vehicle over the course of treatment. Relative to the vehicle, after three and five days of treatment with auranofin, a 1.04 (91%) and 1.40 (96%) average log_10_-reduction of recovered *N*. *gonorrhoeae* was observed. In conclusion, auranofin has the potential to be further investigated as a novel, safe anti-gonococcal agent to help meet the urgent need for new antimicrobial agents for *N*. *gonorrhoeae* infection.

## Introduction

*Neisseria gonorrhoeae* is the causative agent of gonorrhea, which is the second-most reported sexually transmitted disease in the United States. According to the U.S. Centers for Disease Control and Prevention (CDC), more than 616,000 cases of gonorrhea were reported in the U.S in 2019, which represents a 56% increase since 2015 [1]. Furthermore, the World Health Organization (WHO) estimated 87 million new gonorrhea infections occurred worldwide in 2016 [2]. However, the number of reported cases does not represent the true burden of gonorrhea because many infections are asymptomatic.

*N*. *gonorrhoeae* mainly infects the cervix in females and the anterior urethra in males. Symptoms of gonorrhea mainly include purulent discharge and inflammation of the cervix or urethra. Untreated cervical infection may lead to serious complications, such as pelvic inflammatory disease, ectopic pregnancy, and infertility [3, 4]. Pregnant women infected with *N*. *gonorrhoeae* have a high risk of vertical transmission to the fetus/newborn, which may lead to septic abortion and neonatal conjunctivitis [5]. In males, untreated urethritis due to gonorrhea may cause epididymitis or prostatitis. In rare cases, untreated urogenital gonorrhea can disseminate outside the genitals and cause septic arthritis, endocarditis, and meningitis in both males and females [3, 4]. Moreover, *N*. *gonorrhoeae* infection can predispose people to the acquisition and transmission of human immunodeficiency virus infection [6].

Since 1940, gonorrhea has been treated with a range of antibiotics, including sulfonamides, penicillin, tetracycline, and ciprofloxacin. However, the development of resistance by strains of *N*. *gonorrhoeae* to these drugs led to discontinuation of their use [7]. In 2018, treatment recommendations for gonorrhea included ceftriaxone in combination with azithromycin [8]. However, 5.1% of *N*. *gonorrhoeae* isolates had elevated azithromycin minimum inhibitory concentration (MIC) values in 2019 [1]. As a result, in December 2020, the CDC removed azithromycin from the treatment guidelines for gonorrhea. Currently, the CDC recommends a single 500 mg intramuscular dose of ceftriaxone for uncomplicated gonorrhea [9]. Further compounding the problem, *N*. *gonorrhoeae* isolates with reduced susceptibility to ceftriaxone have been reported in the U.S. and worldwide [10–13]. Due to its rapid and progressive development of resistance to currently available antibiotics, *N*. *gonorrhoeae* is classified by the CDC as an urgent threat [14]. Therefore, there is an urgent need for new antibiotics to treat gonococcal infections. Currently, there are only two new antimicrobial agents, zoliflodacin and gepotidacin, that have shown very promising results in Phase 2 clinical trials to treat gonorrhea [15–17]; however, more anti-gonococcal agents are urgently needed.

Auranofin is a gold-containing compound that was approved by the U.S. Food and Drug Administration for the treatment of rheumatoid arthritis. Auranofin has demonstrated excellent antibacterial activity against drug-resistant Gram-positive bacteria, including multidrug-resistant *Staphylococcus aureus*, *Clostridioides difficile*, vancomycin-resistant *Enterococcus faecalis* and *E*. *faecium*, *Staphylococcus epidermidis*, *Streptococcus pneumoniae*, and *Streptococcus agalactiae* [18–25]. Moreover, auranofin possesses antibacterial activity against *Mycobacterium tuberculosis* [26]. Auranofin also demonstrated antimicrobial activity against Gram-positive bacteria in different mouse models, including skin infection, systemic, and peritonitis models [18, 20, 21, 25–27]. Auranofin is considered safe for systemic administration, with no serious side effects or long-term safety concerns [28]. It is now being investigated for the treatment of amebiasis or giardiasis in a Phase II clinical trial (NCT02736968).

Auranofin was previously identified as a promising anti-gonococcal agent in a wide-scale drug repurposing screen using the Prestwick Chemical Library [29]. In addition, in our previous study, we investigated the activity of auranofin against *N*. *gonorrhoeae in vitro* [30].Auranofin exhibited potent antibacterial activity against clinical isolates of *N*. *gonorrhoeae*, including multidrug-resistant strains, as manifested by MIC values that were as low as 0.03 μg/mL. Additionally, a time-kill assay demonstrated that auranofin exhibited rapid bactericidal activity *in vitro* against *N*. *gonorrhoeae*. Moreover, auranofin eradicated intracellular *N*. *gonorrhoeae* in infected endocervical cells (END1/E6E7), outperforming ceftriaxone, the first-line drug [30]. Building upon these promising *in vitro* results, in this study we investigated the *in vivo* activity of auranofin against *N*. *gonorrhoeae* using a mouse model of vaginal infection.

## Materials and methods

### Bacterial strains, chemicals, and media

The FA1090 (ATCC 700825) strain of *N*. *gonorrhoeae* was obtained from the American Type Culture Collection (ATCC) (Manassas, VA). GC agar base, Chocolate II agar, dried bovine hemoglobin, brucella broth and IsoVitaleX were obtained from Becton, Dickinson, and Company (Cockeysville, MD). Heart infusion agar was obtained from Hardy Diagnostics (Santa Maria, CA). Yeast extract and dextrose were obtained from Fisher Bioreagents (Fair Lawn, NJ). Auranofin, hematin, pyridoxal, and nicotinamide adenine dinucleotide (NAD) were obtained from Chem-Impex International (Wood Dale, IL). Protease peptone and VCNT supplement were purchased from Oxoid (Lenexa, KS). Phosphate-buffered saline (PBS) was obtained from Corning (Manassas, VA). Ceftriaxone, azithromycin and saponin were obtained from TCI America (Portland, OR) Tween 80 was obtained from Acros Organics (Fair Lawn, NJ). Estradiol pellets (5-mg, 21-day controlled-release) were purchased from Innovative Research of America (Sarasota, FL). Dacron swabs were purchased from the Medical Packaging Corporation (Camarillo, CA).

### Minimum inhibitory concentration

The MIC values of auranofin, azithromycin, and ceftriaxone against *N*. *gonorrhoeae* FA1090 were determined, as described previously [31–34]. Briefly, a bacterial suspension equivalent to a McFarland standard of 1.0 was prepared and diluted in Brucella Broth supplemented with yeast extract, dextrose, proteose-peptone, NAD, pyridoxal, and hematin to obtain a bacterial count of 1 × 10^6^ CFU/mL. Diluted bacteria were incubated with serial dilutions of auranofin, azithromycin, or ceftriaxone at 37°C for 24 hours in the presence of 5% CO_2_.

### Evaluating the activity of auranofin against *N*. *gonorrhoeae* in mice

#### Preparation of *N*. *gonorrhoeae* for the animal study

*N*. *gonorrhoeae* FA1090 was streaked on GC agar plates and incubated at 37°C for 20 hours in the presence of 5% CO_2_. Separate colonies were used to prepare a bacterial suspension in sterile PBS. The bacterial suspension was filtered to remove bacterial aggregates and then diluted with PBS to reach a concentration of ~10^8^ CFU/mL. The bacterial count was confirmed by dilution and plating onto GC agar plates.

#### Mouse model of *N*. *gonorrhoeae* vaginal infection

Animal experiments were reviewed, approved, and performed under the guidelines of Virginia Polytechnic Institute and State University Institutional Animal Care and Use Committee (IACUC). Animal experiments were carried out in strict accordance with the recommendations in the Guide for the Care and Use of Laboratory Animals of the National Institutes of Health. The animal studies are in compliance with the Animal Research: Reporting of In Vivo Experiments (ARRIVE) guidelines. According to the approved animal protocol, if any animal meets more than two of the group I criteria for 48 hours with no improvement, it will be closely monitored and a veterinarian will be consulted to determine how to proceed (euthanize, treat or continue to monitor). If any animal exhibits any one of the group II criteria, it will be euthanized promptly. Group I Criteria: a. rough coat and unkempt b. Eyes full or partially closed for 10 min c. Markedly diminished resistance to being handled (grimace response) d. Markedly decreased movement/lethargy e. hunched posture F. distended abdomen. Group II Criteria: a. Inability to eat or drink b. moribund/unresponsive c. Failure to right self when placed on back d. Dyspnea e. 15% or more loss in the body weight. A total of 30 animals were used in the study and all of them were euthanized at the end of experiment using carbon dioxide. No humane end point was reached. The duration of the study was 10 days. Animal health and behavior were monitored twice daily. Animal welfare considerations were taken, including efforts to minimize suffering and distress. Isoflurane was given by inhalation to anaesthetize mice during the steps that are associated with pain (vaginal swab or using the trochar). Research staff had all the necessary training including animal injection, euthanasia, oral gavage and the use of trochar.

**Fig 1:**
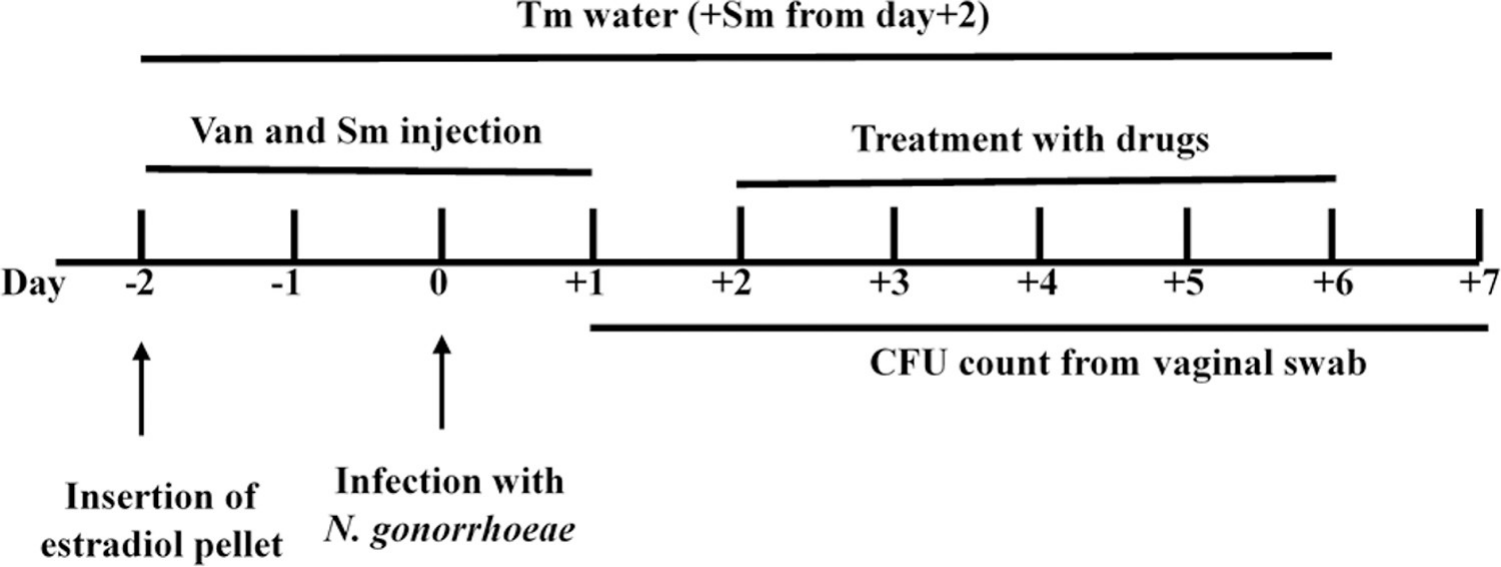
Schedule of the infection, treatment, and sample collection of *N*. *gonorrhoeae* in the female mouse model of genital tract infection. Two days before the infection, 8-week-old female ovariectomized BALB/c mice were implanted subcutaneously with a 5-mg, 21-day controlled-release estradiol pellet. Mice were given antibiotics (Tm, trimethoprim; Sm, streptomycin; Van, vancomycin) throughout the experiment, as described in the Materials and Methods section. Two days following vaginal infection, mice were allocated into groups and orally administered either auranofin (0.25 mg/kg) or vehicle (10% DMSO, 10% Tween 80, 80% PBS) for five days. One group of mice was given a single intraperitoneal dose of ceftriaxone (15 mg/kg) as a positive control. Vaginal swabs were collected daily. Samples were serially diluted and plated on GC agar supplemented with vancomycin, colistin, nystatin, and trimethoprim (VCNT).

*N*. *gonorrhoeae* infection was established in the vagina of mice, as described previously by Raterman and Jerse [35]. Fig 1 presents a schematic of the mice study. Briefly, female 8-week-old ovariectomized BALB/c mice (Jackson Laboratories, Bar Harbor, ME) were anesthetized using isoflurane inhalation and shaved. Mice were subsequently implanted with 5-mg, 21-day controlled-release estradiol pellets subcutaneously using precision trocars (Innovative Research of America, Sarasota, FL), and the incision was glued using 3M Vetbond Tissue Adhesive (3M, Saint Paul, MN). Two days after pellet implantation, mice were inoculated intravaginally with 2.3 × 10^6^ CFU of *N*. *gonorrhoeae* FA1090. Mice were injected intraperitonially with 4 mg/L of vancomycin and 24.0 mg/L of streptomycin on Days −2 through +1. Drinking water was replaced on Day −2 with water containing 0.4 mg/L trimethoprim. Trimethoprim-containing water was refreshed every other day for the duration of the experiment. Streptomycin sulfate (2.4 mg/L) was added to the water after Day +1. Two days post-infection, mice were randomly allocated into groups (n = 10) and administered either auranofin (0.25 mg/kg) or vehicle (10% DMSO, 10% Tween 80, 80% PBS) via the oral route for five consecutive days. As a positive control, one group of mice received a single intraperitoneal dose of ceftriaxone (15 mg/kg). *N*. *gonorrhoeae* vaginal colonization was quantified by collecting vaginal swabs daily. Moistened Dacron swabs were gently inserted into the vagina of anaesthetized mice and then suspended into 0.1 mL of GC broth containing 0.05% saponin. Samples were serially diluted and plated on GC agar supplemented with VCNT (contains 600 μg of vancomycin, 1.5 mg of colistin, 2500 units of nystatin, and 1 mg of trimethoprim lactate per liter). To monitor the presence of commensal flora that could potentially inhibit the growth of *N*. *gonorrhoeae*, vaginal swabs were streaked on heart infusion agar and the resulting growth was Gram stained. Contaminated samples were excluded from the experiment.

### Statistical analyses

Data were analyzed via two-way ANOVA with post-hoc Dunnett’s test for multiple comparisons, utilizing GraphPad Prism version 8 for Windows (GraphPad Software, La Jolla, CA).

## Results and discussion

The *in vitro* antibacterial activity of auranofin against *N*. *gonorrhoeae* FA1090 (ATCC 700825) was determined. Consistent with our previous study, auranofin displayed potent anti-gonococcal activity and inhibited the growth of *N*. *gonorrhoeae* FA1090 at a concentration of 0.125 μg/mL. Azithromycin and ceftriaxone were used as positive controls. They inhibited the growth of the tested *N*. *gonorrhoeae* strain at a concentration of 0.06 μg/mL (azithromycin) and 0.002 μg/mL (ceftriaxone), respectively.

The *in vivo* activity of auranofin against *N*. *gonorrhoeae* FA1090 was evaluated using a female mouse model of *N*. *gonorrhoeae* genital tract infection, as previously described [35]. This model has been successfully used to study host-pathogen interactions and the genetic basis for antibiotic resistance [36–39]. Importantly, this model permits the testing of antimicrobial agents, immunotherapies, and vaccines against *N*. *gonorrhoeae* infection [40–45]. It has been established that *N*. *gonorrhoeae* can colonize the female genital tract of mice when inoculated into the vagina during the diestrus or anestrus stages of the estrous cycle [46]. The duration of infection with *N*. *gonorrhoeae* can be prolonged via treatment of mice with 17β-estradiol [47].

In this study, we established an *N*. *gonorrhoeae* infection in female ovariectomized BALB/c mice that were subcutaneously implanted with a 5-mg, 21-day controlled-release estradiol pellet two days before the infection (Fig 1). Ovariectomized mice were used because they do not need to be staged prior to estradiol treatment. On the other hand, intact (regular) mice require staging (two times, three days apart) to identify the mice in the proper stage (diestrus or anestrus). Thus, if using regular mice, the total number of animals should be increased, as some mice may not be in the correct stage to establish *N*. *gonorrhoeae* infection. Additionally, the recovery of *N*. *gonorrhoeae* from ovariectomized mice is better than that from intact mice, because there are no observed fluctuations in the number of recovered *N*. *gonorrhoeae* from ovariectomized mice [35, 36]. Thus, the use of ovariectomized mice in our study eliminated the unnecessary use of extra mice and improved the recovery of *N*. *gonorrhoeae*. In this study, mice were administered an antibiotic cocktail throughout the experiment to avoid the overgrowth of vaginal commensal flora that could inhibit *N*. *gonorrhoeae* infection (Fig 1). The overgrowth of vaginal microbiota can be due to estrogen-induced proliferation of vaginal cells that result in elevated levels of free glycogen [35]. The establishment of *N*. *gonorrhoeae* infection and the effect of different treatments on *N*. *gonorrhoeae* colonization of the vagina of mice were tested by collecting vaginal swabs daily and plating on GC agar containing vancomycin, colistin, nystatin, and trimethoprim (VCNT) supplement to select for the growth of *N*. *gonorrhoeae*.

**Fig 2:**
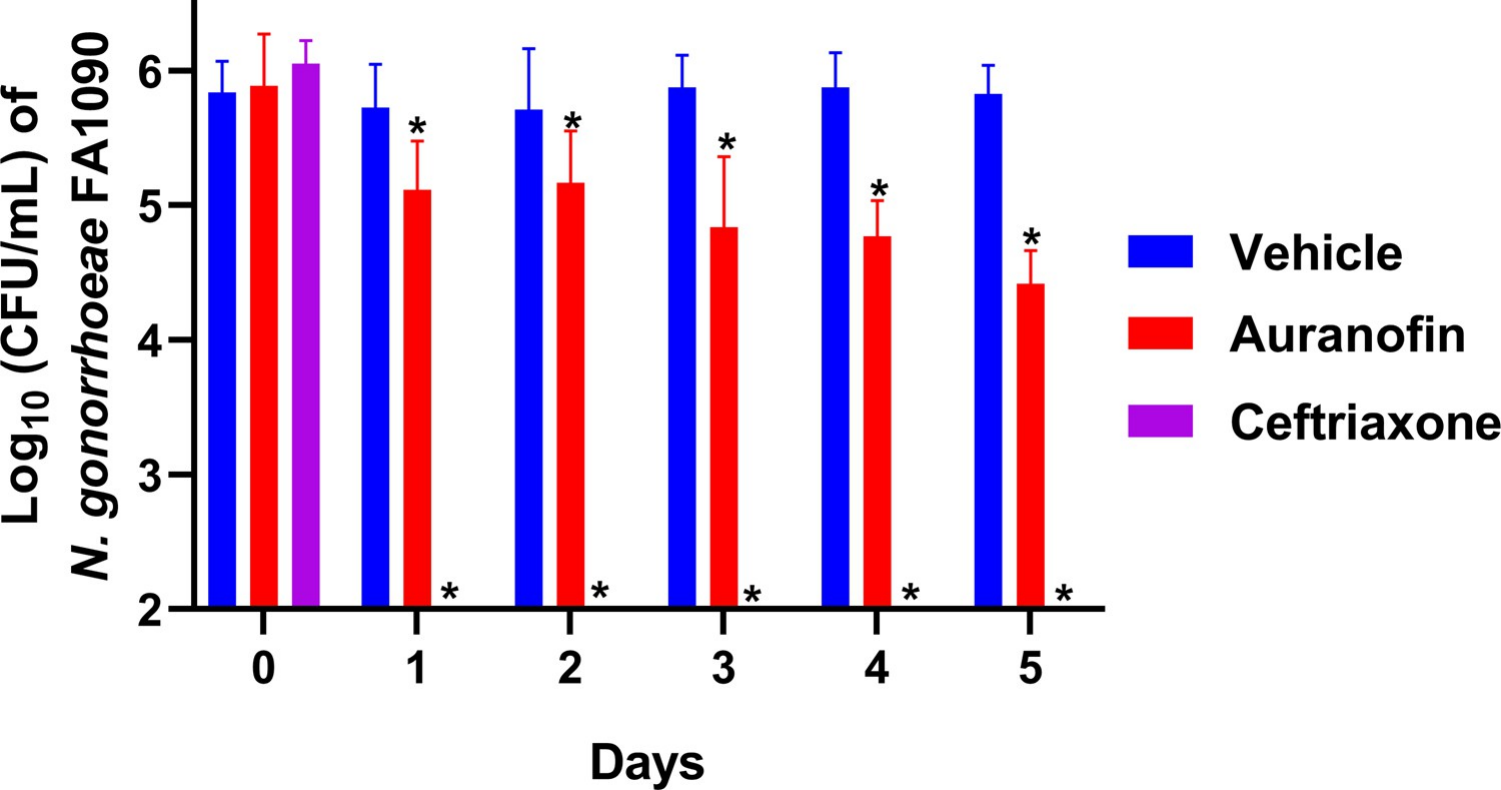
Viable count of *Neisseria gonorrhoeae* (average ± standard deviation log_10_ CFU/mL) recovered from vaginal swab of mice over the course of five days of treatment with vehicle, auranofin, or ceftriaxone. Female ovariectomized BALB/c mice that were subcutaneously implanted with 5-mg, 21-day controlled-release estradiol pellets were infected intravaginally with *N*. *gonorrhoeae* FA1090 and then were treated orally with auranofin (0.25 mg/kg) or vehicle (10% DMSO:10% Tween 80:80% PBS) for five consecutive days. As a positive control, a group of mice received a single intraperitoneal dose of ceftriaxone (15 mg/kg). Vaginal swabs were collected daily and cultured to determine the number of viable bacteria colonizing the vagina of mice. The data were analyzed via a two-way ANOVA with post-hoc Dunnett’s test for multiple comparisons. An asterisk (*) indicates a significant difference (*P* <0.05) between mice treated with auranofin or ceftriaxone compared with the vehicle.

Mice were treated with auranofin (0.25 mg/kg) orally for five consecutive days. Auranofin was delivered via the oral route because this is the normal route of administration for humans [48]. As shown in Fig 2, treatment of mice with auranofin significantly reduced the number of recovered *N*. *gonorrhoeae* compared to vehicle control over the course of treatment. After three days of treatment, auranofin reduced the vaginal burden of *N*. *gonorrhoeae* by 1.04-log_10_ (91%) relative to the vehicle control. The gonococcal burden continued to significantly decrease further with auranofin treatment, resulting in a 1.41-log_10_ (96%) reduction of *N*. *gonorrhoeae* recovered from vaginal samples after five days of treatment. However, none of the mice treated with auranofin cleared the infection after five days of treatment. Mice treated with a single dose of ceftriaxone cleared the infection within 24 hours of treatment.

Previous studies have shown that auranofin has a good safety profile [28]. Chronic exposure of patients to auranofin over extended periods of time was safe with no cumulative toxicity observed over five years [28].The usual adult dosage of auranofin is 6–9 mg daily for up to six months, which is a much longer course of treatment than what would be expected for auranofin as an antibacterial agent [20, 49]. Consequently, the dose used in this study, (0.25 mg/kg), is within range of clinically-administered human doses. In the present study, auranofin-treated mice, as expected, showed no weight loss or other obvious comorbidities. Therefore, auranofin is a promising, safe drug to be administered alone or in combination with other antibiotics to treat gonorrheal infections.

In our previous study, auranofin showed excellent activity against *N*. *gonorrhoeae* strains *in vitro* as demonstrated by MIC values and a time-kill assay [30]. Additionally, auranofin outperformed ceftriaxone in eliminating intracellular *N*. *gonorrhoeae* infection of endocervical cells (END1/E6E7) [30]. In this study, mice treated with auranofin at a dose of 0.25 mg/kg showed an average reduction in gonococcal burden by 1.4-log_10_ (96%) relative to the vehicle after five days of treatment. It is tempting to hypothesize that increasing the dose of auranofin or the duration of treatment may result in a much better anti-gonococcal effect in mice. Additionally, since gonococcal infections have previously been treated with a combination of two drugs, azithromycin and ceftriaxone, we propose that the combination of auranofin with azithromycin or ceftriaxone may possess a synergistic effect against *N*. *gonorrhoeae in vivo*. However, this must be investigated in a future study.

## Supporting information

S1 Data Minimal underlying data set. (XLSX)
